# Transepithelial Transport Characteristics of the Cholesterol- Lowing Soybean Peptide, WGAPSL, in Caco-2 Cell Monolayers

**DOI:** 10.3390/molecules24152843

**Published:** 2019-08-05

**Authors:** Huijuan Zhang, Yawen Duan, Yulin Feng, Jing Wang

**Affiliations:** 1 Innovation Center for Food Nutrition and Human Health, Beijing Technology & Business University (BTBU), Beijing 100048, China; 2 Beijing Engineering and Technology Research Center of Food Additives, Beijing Technology & Business University (BTBU), Beijing 100048, China

**Keywords:** soybean protein, WGAPSL, transport, Caco-2 cells

## Abstract

Recent studies have shown that soybean protein and its peptides have cholesterol-lowering activities. However, it is not clear whether these peptides could overcome physiological barriers, such as phase II metabolism in gastrointestinal tract and poor permeability, to reach the blood stream in its intact form. Therefore, the transepithelial transport characteristics of soybean peptide Trp-Gly-Ala-Pro-Ser-Leu (WGAPSL) with cholesterol- lowering activity were investigated in Caco-2 cells. In this study; the transepithelial absorption of WGAPSL was studied using human intestinal Caco-2 cell monolayers. The results showed that WGAPSL had good stability (83.9% ±1.98%) after simulated gastric and intestinal digestion. During the apical (AP) side to basolateral (BL) side transport, WGAPSL was absorbed intact through Caco-2 cell monolayers with apparent permeability coefficient (Papp) values of 4.4 × 10^−8^ to 1.2 × 10^−8^ cm/s. Cytochalasin D loosened the tight junctions of Caco-2 cell monolayers and significantly (*p* < 0.05) improved the transport process. Sodium azide, wortmannin, and Gly-Pro had minimal effects on transport, demonstrating that the major transport route of WGAPVL was paracellular via tight junctions. Finally, LC-MS analysis showed that Gly-Ala-Pro (GAP) was the important part for the intact absorption of WGAPVL and Trp (W) was the most unstable amino acid residue.

## 1. Introduction

After oral administration, food-derived peptides need to overcome two important physiological barriers to be absorbed intact into blood circulation—extensive phase II metabolism in the gastrointestinal tract and poor permeability through the intestinal epithelium [1]. Therefore, bioavailability is the limiting factor for the application of food-derived peptides. Caco-2 cell monolayer, a human intestinal carcinoma cell line, simulates the human gut in terms of biological functions and the microvilli structure, and resembles tight junctions. Hence, it has been used to test the absorption mechanism of drugs across the intestinal epithelium [2,3]. The distinctive features determining the potential of peptides to be transported intact are hydrophobicity, charge, molecular mass, and tendency to aggregate [4,5].

Some oligopeptides can be absorbed by Caco-2 cell monolayers intact. A recent study has reported that tight junctions were the major transport mechanism for Gln-Ile-Gly-Leu-Phe (QIGLF), a peptide derived from egg white ovalbumin with angiotensin converting enzyme (ACE)-inhibitory activity, across Caco-2 cell monolayers [6]. The transport of the peptide Val-Leu-Pro-Val-Pro (VLPVP), investigated by DNA recombinant technology using an efficient *Escherichia coli* expression system, involves paracellular diffusion, and multidrug resistance-associated protein 2 (MRP2) was reported as the main transporter [7]. In contrast, a β-casein (193–209) peptide resisted the digestion of brush border membrane peptidases, and its main transport route was transcytosis via internalized vesicles on Caco-2 cell monolayers [8]. The absorbance patterns of anserine and carnosine in Caco-2 cell monolayers depend on their coactions with the human intestinal peptide transporters 1 and 2, which were transported by human H^+^⁄peptide cotransporters (hPepT1), a main transport system for di-peptides and tri-peptides [9]. Furthermore, it has been shown that the predominant route involved in the transepithelial efflux of Tyr-Pro-Ile (YPI), derived from egg proteins, is probably PepT1 [10]. An intracellular pathway termed adsorptive transcytosis was suggested to transport bradykinin and its analogues, and was mainly associated with the peptides’ hydrophobic properties [11].

The effects of soybean protein (SP) and its hydrolysates on cholesterol and bile acid metabolism have been investigated in previous studies [12,13,14]. Compared with casein tryptic hydrolysate (CTH), SP peptic hydrolysates with bound phospholipids efficiently lowered micellar cholesterol solubility in Caco-2 cell monolayers [15]. Ovomucin had an apparently greater bile acid binding ability than casein in Caco-2 cell monolayers [16]. The hypocholesterolemic activities of soybean protein hydrolysates (Degree of Hydrolysis18%) were confirmed in a high fat diet mice model [17], while the sequence of bioactive peptide, WGAPSL, was determined using liquid chromatography–mass spectrometry (LC-MS) and reverse phase–high-performance LC (RP-HPLC) [18]. Moreover, WGAPSL increased the very low density lipoprotein-cholesterol (VLDL-C) and triglyceride (TG) contents and decreased the low density lipoprotein-cholesterol (LDL-C) content in mice [19]. However, the transepithelial transport characteristics of this peptide are still obscure. Therefore, the present study investigated the sensitivity of WGAPSL to hydrolysis by brush border enzymes and its transepithelial transport mechanism across Caco-2 cell monolayers. The transepithelial transport pathway of WGAPSL was determined using selective inhibitors of different transport mechanisms. 

## 2. Results and Discussion

### 2.1. Cytotoxicity in Caco-2 Cells

**Figure 1:**
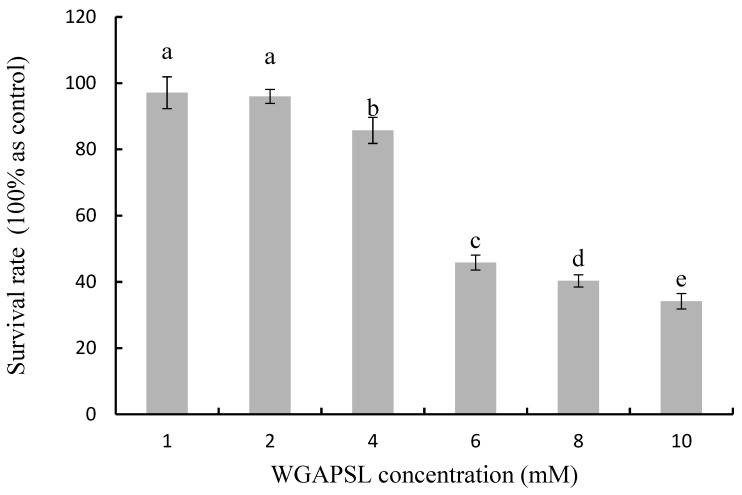
Cytotoxicity of Trp-Gly-Ala-Pro-Ser-Leu (WGAPSL) in Caco-2 cells as evaluated by the CCK-8 assay. Data are the mean values ± standard variation of three replications. Different letters (a, b, c, and d) indicate a significant difference at *p* < 0.05.

The Cell Counting Kit-8 (CCK-8) assay demonstrated that the WGAPSL peptide was non-toxic for Caco-2 cells at a concentration of 1 mM and 2 mM (dissolved in Dulbecco’s Modified Eagle Medium, DMEM, Figure 1). However, higher WGAPSL concentrations showed significant cytotoxic effects on Caco-2 cells. At 10 mM, the survival rates decreased to 27%. Therefore, the optimal concentration of 2 mM was used in the following experiments.

### 2.2. Peptides Stability

**Figure 2:**
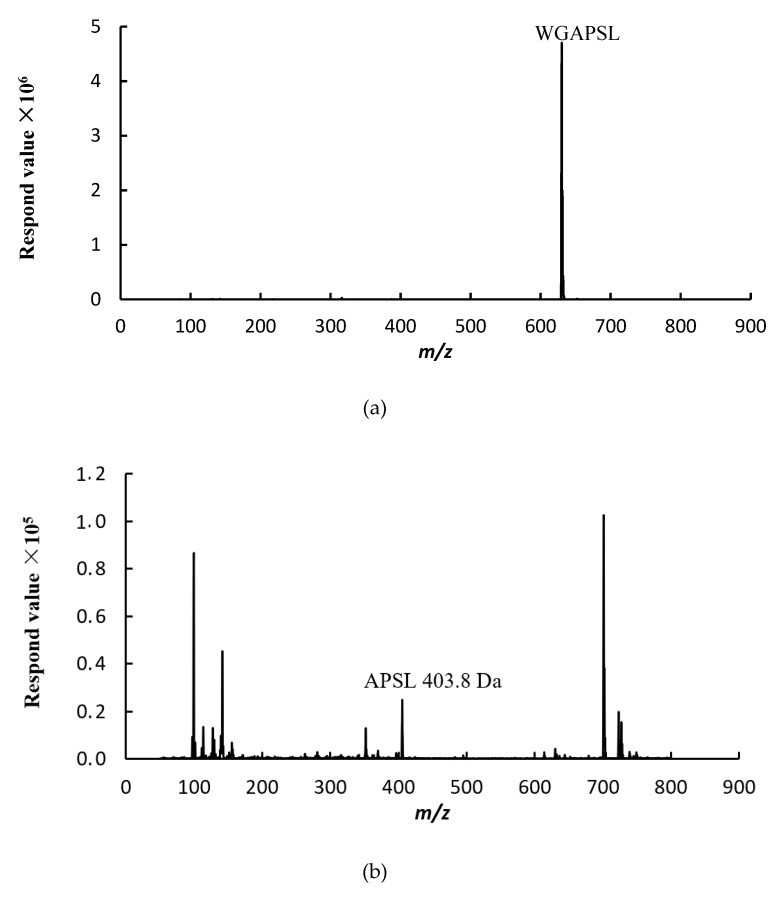

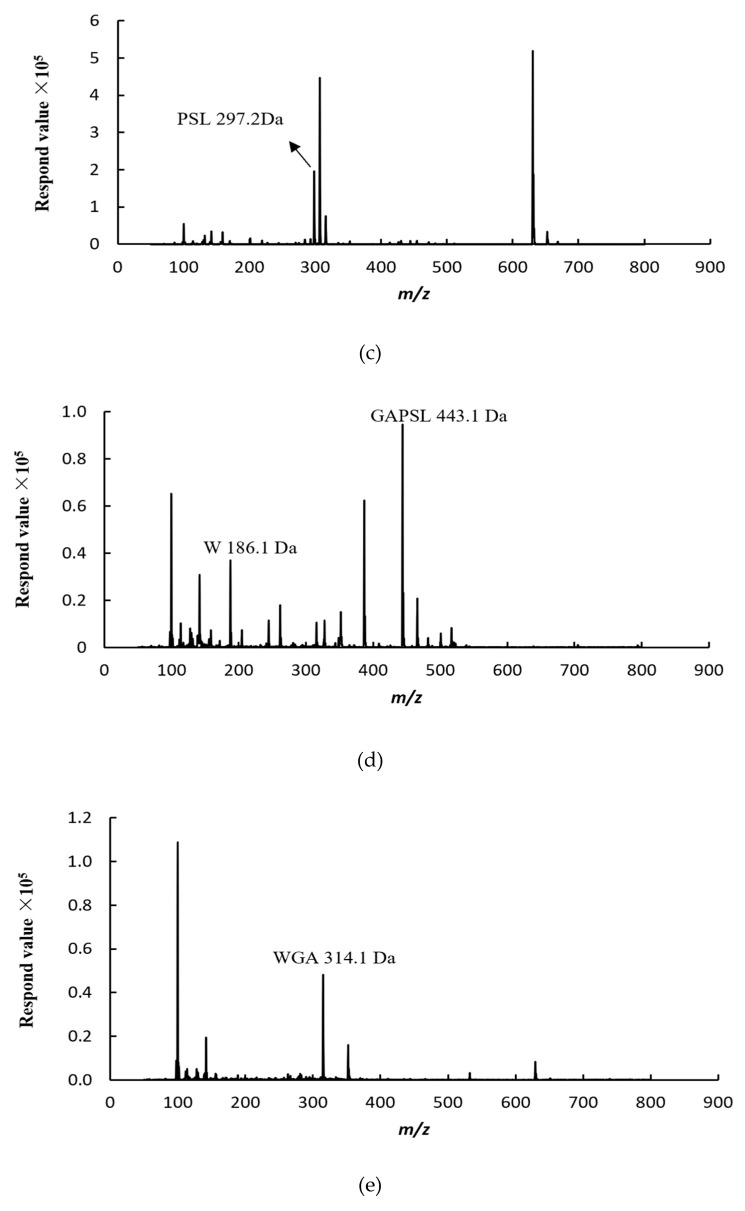

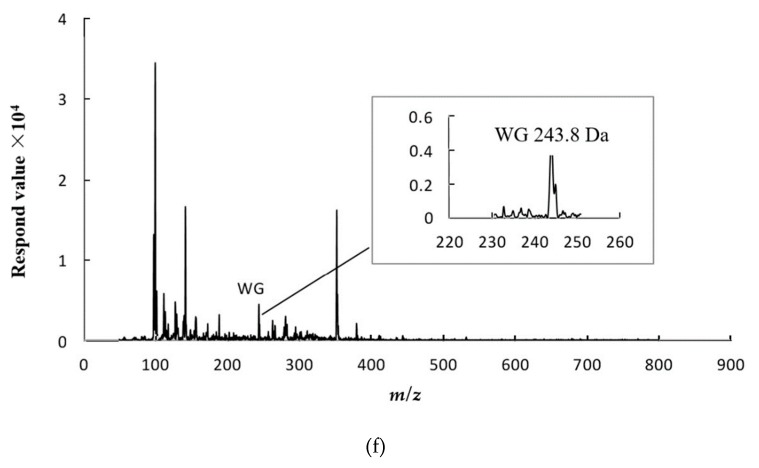
Liquid chromatography–mass spectrometry (LC-MS) spectrogram of peptide fragments of WGAPSL after simulated gastrointestinal digestion. (**a**) Intact WGAPSL; (**b**) fragment APSL; (**c**) fragment PSL; (**d**) fragment W and GAPSL; (**e**) fragment WGA; (**f**) fragment WG.

A two-stage hydrolysis process, which simulated in vivo conditions during physiological digestion, was carried out to evaluate the resistance of WGAPSL identified in soybean to digestive enzymes (Figure 2). The results show that WGAPSL had good stability (83.9% ± 1.98%) after the simulation of in vitro digestion. The “Keil rule” that trypsin cleaves next to arginine or lysine, but not before proline, has been generally accepted [20,21,22]. Therefore, the good stability of WGAPSL might be because of the proline residue making sequences less susceptible to proteolytic enzymes. Figure 2 also shows that APSL, PSL, W, GAPSL, WGA, and WG were the fragments hydrolyzed by the gastrointestinal enzymes. The WGAPSL degradation mechanism in gastrointestinal digestions was mediated via the trypsin cleavage sites, such as Trp (W), Leu (L), and so on [21,22].

### 2.3. Transepithelial Absorption of WGAPSL through Cell Culture Model

**Figure 3:**
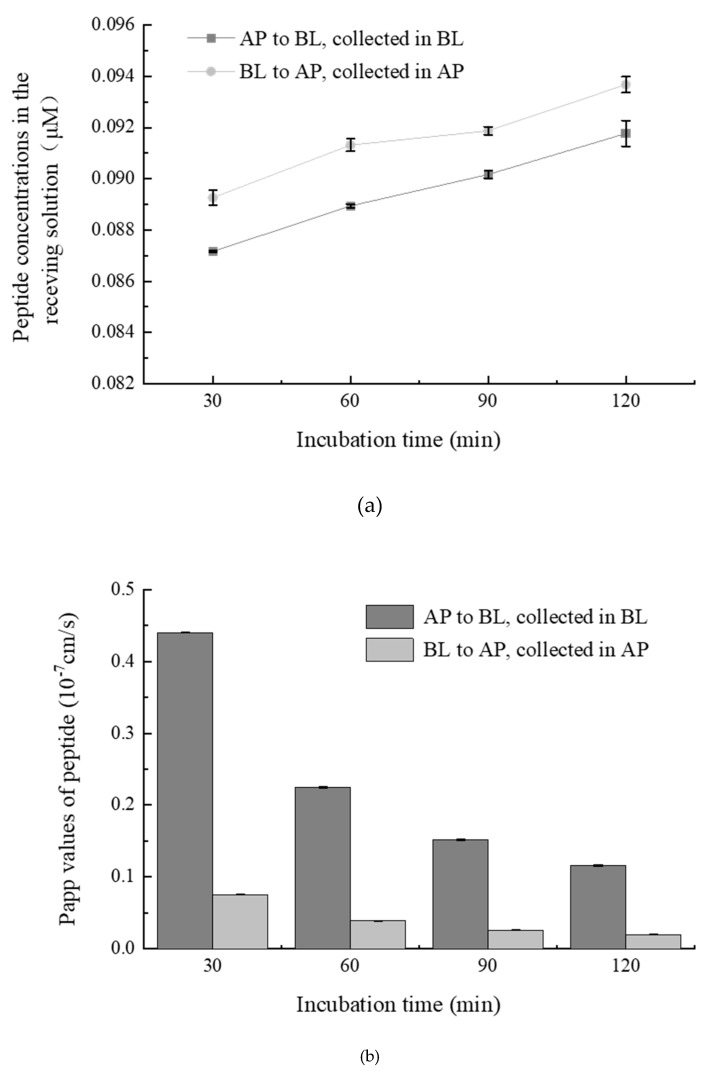
(**a**) Time-course of WGAPSL (2 mM, dissolved in hank’s balanced salt solution, HBSS, pH 7.4) transport across the Caco-2 cell monolayers from apical (AP) to basolateral (BL)and from BL to AP. (**b**) Papp (apparent permeability coefficient) values of peptides WGAPSL transport from AP to BL and collected in BL, and transport from BL to AP and collected in AP. Data are mean values ± standard variation of three replications and different letters indicated significant difference at *p* < 0.05.

The time-dependent uptake of WGAPSL across Caco-2 cells from the apical (AP) side to the basolateral (BL) side and from BL to AP is shown in Figure 3. During the AP→BL and BL→AP transport experiments, the WGAPVL amount in the receiving solution significantly (*p* < 0.05) increased to 0.091 µM and 0.094 µM at 120 min (Figure 3a). The apparent permeability coefficient (Papp) value reflects the velocity of passage through the epithelium. During the AP→BL transport experiment, the Papp values of WGAPVL from 30 min to 120 min were from 4.4 × 10^−8^ cm/s to 1.2 × 10^−8^ cm/s (Figure 3b). Some studies have reported that the Papp value of casoxin-6 was 9.21 × 10^−6^ cm/s, while the Papp value of Val-Pro-Pro (VPP) was 0.5 × 10^−8^ cm/s [23,24]. The Papp value presented similar magnitudes in both directions. The Papp values of the peptide decreased by about 64.2% from 30–120 min during the AP→BL flux, while they decreased by about 65.6% during the BL→AP transport experiment. 

To analyze the possible role of the peptide transport pathway, the efflux ratio (ER) of WGAPVL was calculated. The ER values of WGAPSL from 30–120 min were 0.169–0.170, indicating that WGAPSL was not a substrate of P-glycoprotein and it required a concentration-dependent absorption flux with no adenosine triphosphate (ATP) involvement. 

### 2.4. Effects of Various Inhibitors on Peptide Transport

**Figure 4:**
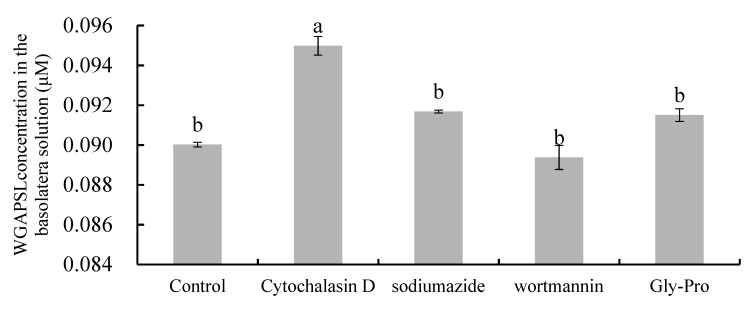
Effects of transport pathway inhibitors on the transport of WGAPSL in Caco-2 cell monolayers. Two mM WGAPSL (dissolved in Hanks, pH 7.4) was added to the AP side following preincubation with four transport inhibitors (cytochalasin D, sodium azide, wortmannin, and Gly-Pro) for 30 min. Different letters indicate a significant difference at *p* < 0.05.

To elucidate the transepithelial transport pathway of WGAPSL, some inhibitors, such as cytochalasin D, wortmannin, sodium azide, and Gly-Pro, were added to Caco-2 cell monolayers (Figure 4). There are four gradients transport routes in Caco-2 cells: passive diffusion, paracellular, transcytosis, and endocytosis [25]. Transcellular passive diffusion is mainly used for hydrophobic substances, while paracellular transport is used for water-soluble, low-molecular weight substances [6]. Cytochalasin D, a tight junctions disruptor, significantly (*p* < 0.05) increased the AP→BL flux of WGAPSL and its concentration in the BL side increased by 66% compared with the control group (Figure 4). This result demonstrates that this peptide was transepithelially transported through the small intestinal epithelium, possibly mainly via the paracellular pathway. The Papp value of QIGLF, an ACE-inhibitory peptide from egg white, significantly (*p* < 0.05) increased after cytochalasin D treatment [6], while a bovine β-casein 17-residues peptide (β-CN193-209) was mainly transported by transcytosis, owing to its large molecular weight and hydrophobic properties [8]. To determine whether the transepithelial transport of this peptide is carrier-mediated, a competitive substrate for peptide transporter (Gly-Pro) was used. However, the transport of the peptide was not significantly (*p* > 0.05) decreased by 10 mM Gly-Pro, demonstrating that the transporter PepT1 was not involved in the transport of WGAPSL across Caco-2 cells. The reason for this may be because PepT1, an active and saturable symporter, is mainly responsible for the transepithelial transport of many di- and tri-peptides, such as Ile-Arg-Try (IRW) and Tyr-Pro-Ile (YPI) [10,26]. A similar result was observed upon pre-incubation with 10 mM sodium azide, indicating that the transport of WGAPSL was energy-independent. Moreover, the addition of 500 nM wortmannin, a transcytosis inhibitor, did not significantly (*p* > 0.05) affect the concentration of the peptide, suggesting that it was not transported by transcytosis. Studies of the transport of some oligopeptides, such as Val-Leu-Pro-Val-Leu (VLPVL), Lys-Val-Leu-Pro-Val-Pro (KVLPVP), His-Leu-Pro-Leu-Pro (HLPLP), and Gln-Ile-Gly-Leu-Phe (QIGLF), showed that the paracellular pathway was the primary transport route [6,7,27,28].

### 2.5. Peptide Degradation Fragments in Caco-2 Cell Monolayers

**Figure 5:**
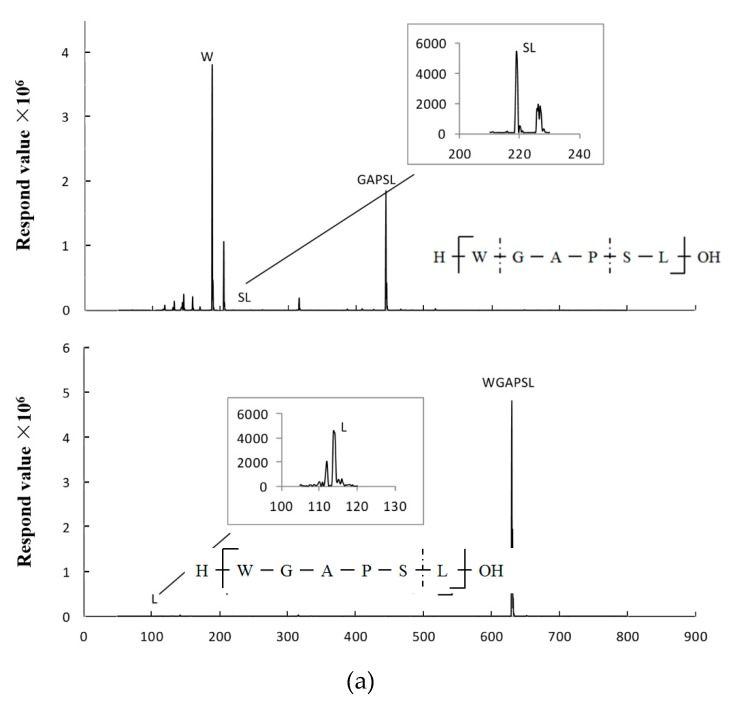

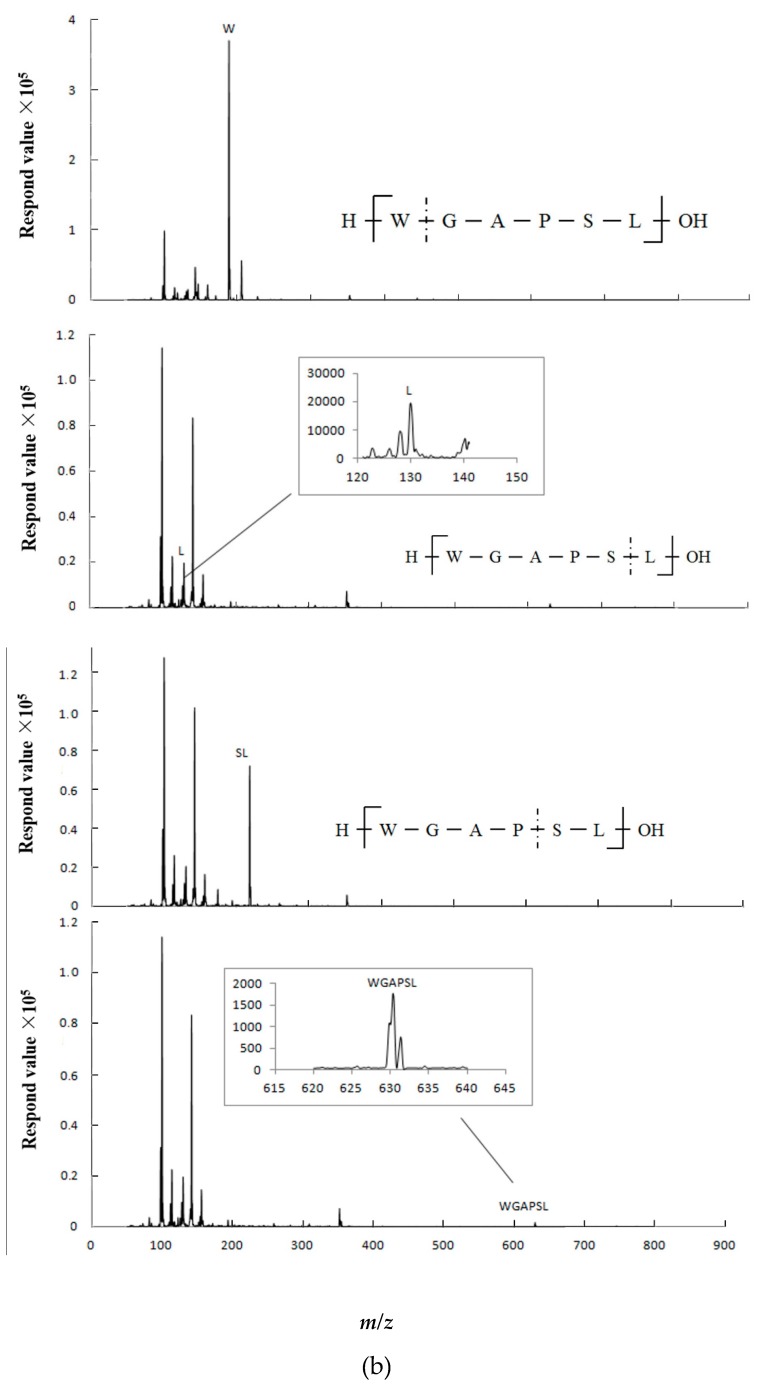
Fragments detected by LC-MS in apical (AP) (**a**) and basolateral (BL) (**b**) sides after the incubation of peptide WGAPSL in Caco-2 cell monolayers for 120 min. (Trp: W, Leu: L, and Ser-Leu: S-L).

Brush border peptidases may be the main factors that limit the half-life of bioactive peptides in the intestine [29]. Therefore, the resistance of WGAPSL to intestinal peptidases and the digestion fragments were examined using LC-MS analysis. The peptide sequence detected by MS-Scan at 120 min are shown in Figure 5. Among the fragments found in the apical chamber, the intact peptide, Trp (W), Leu (L), and Ser-Leu (S-L) were also transported to the BL chamber. The intact peptide, WGAPSL, shorter peptides, and amino acid residues crossed the intestinal barrier up to the basal side, possibly because of their special chemical and structural properties. To better understand the transport mechanism, the different distributions of peptide degradation fragments on both sides of the small intestinal model were analyzed. According to the peak area, the AP side had higher concentrations of degraded fragments of WGAPSL than the BL side. The most unstable amino acid residue was Trp (W) [19], and the amino acid sequence GAP played an important role in intact peptide absorption. The key amino acid residue determining the intact absorption of WGAPSL might be Pro (P), which is present at the C-terminus of a large number of peptides [30,31].

## 3. Materials and Methods

### 3.1. Materials and Reagents 

Minimal essential medium (MEM) and DMEM were purchased from GE Healthcare Life Science (South Logan, UT, USA). Fetal bovine serum was from PAN Biotech Gmbh (Aidenbach, German). Phosphate buffered saline (PBS), penicillin-streptomycin, HBSS, 4-(2-hydroxyethyl)-1-piperazineethanesulfonic acid (HEPES) buffer, and nonessential amino acids were purchased from Solarbio Co., Ltd. (Beijing, China). Pepsin was purchased from Sigma-Aldrich (St. Louis, MO, USA) and trypsin was purchased from Amerco Inc (Cochran Solon, OH, USA). The cholesterol lowering peptide WGAPSL (90% purity) was synthesized by Ziyubio Co., Ltd. (Shanghai, China) using a solid-phase procedure. 

### 3.2. Caco-2 Cells Culture 

Caco-2 cells were obtained from China Infrastructure of Cell Line Resource (Beijing, China) and were cultured based on the method of Zhang et al. [32]. Cells were cultured in MEM containing 10% fetal bovine serum, 1 mM nonessential amino acid solution, 1% penicillin-streptomycin, and 1% HEPES buffer. Caco-2 cells were seeded at 5 × 10^5^ cells/mL in 25 cm^2^ tissue culture flasks, and incubated at 37 °C in 5% CO_2_ with 95% humidity. The passage number of the cells used in the study was between 30 and 40. The medium in cell cultures were replaced every two days and detached from 70–80% confluency using 0.05% trypsin and 0.02% Ethylene Diamine Tetraacetic Acid (EDTA) solution. For the transport experiments, Caco-2 cells were seeded into a collagen-coated transwell plate (6.5 mm diameter, 0.4 μm pore size Corning Costar) at a density of 1 × 10^5^ cells/well.

### 3.3. Cytotoxicity Assay

The effects of the WGAPSL peptide on Caco-2 cell proliferation were observed using the CCK-8 cell counting kit (Dojindo, Japan). The WST-8 (2-(2-methoxy-4-nitrophenyl)-3-(4-nitrophenyl)-5-(2,4-disulfophenyl)-2*H*-tetrazolium, monosodium salt) reagent was used based on the kit specifications. Caco-2 cells were plated in 96-well culture plates in a final volume of 100 μL/well of culture medium (1 × 10^5^ cells/well) at 37 °C in 5% CO_2_ for 24 h and incubated with 1–10 mM WGAPSL (diluted in DMEM) or DMEM only for 2 h, followed by incubation with 10 μL CCK-8 and incubation for another 4 h and then the absorbance was measured. 

### 3.4. Simulated Gastrointestinal Digestion

Simulated gastrointestinal digestion was done based on the method of Quirós et al. [33]. Synthetic peptide (1 mg/mL) was prepared in HBSS and acidified with HCl to reach pH 2.0, then hydrolyzed with 1:10,000 pepsin at an enzyme/substrate ratio of 1:50 (w/w), and incubated in constant temperature water oscillation at 37 °C for 90 min with a low shaking rate (120 rpm). After gastric digestion, samples were adjusted to pH 7.5 with saturated NaHCO_3_ and 1:250 trypsin was added at an enzyme/substrate ratio of 1:50 (w/w), and incubated in constant temperature water oscillation at 37 °C for 4 h with a low shaking rate (120 rpm). Reactions were stopped in a boiling water bath for 10 min. Digested samples were centrifuged (3K15, SIGMA Laborzentrifugen GmbH, Germany) at 30,000× *g* at 4 °C for 20 min and diluted 100 times to analyze peptide concentrations using liquid chromatography-tandem mass spectrometry (LC-MS, 1200, Agilent, CA, USA).

### 3.5. Transport Experiments

Prior to transport experiments, Caco-2 cell monolayers were gently rinsed with HBSS, and then incubated for 30 min at 37 °C in 5% CO_2_. Afterwards, HBSS was drawn from both the AP side and BL side of all wells. HBSS (0.1 mL) with 2 mM WGAPSL was added to the AP side and 0.6 mL of fresh HBSS was added to the BL side. All transwell plates were incubated at 37 °C in 5% CO_2_ for 30, 60, 90, or 120 min. The transport experiment was done in triplicate for three consecutive days after 21 days, in which the cell model was developed. 

The bilateral transport experiments also assessed transport from the BL to AP side. The mechanisms of transportation were predicted by calculating the Papp value (cm/s) and the ratios of appearance rates/initial concentrations (2 mM) in the donor compartment.

The Papp values were calculated as follows (Equation (1)):

(1) $$\mathrm{Papp}=\frac{dQ}{dt}\times \frac{1}{A}\times \frac{1}{C_{0}}$$

where *dQ*/*dt* is the permeability rate (mmol/s); *A* is the membrane area (cm^2^); and *C*_0_ is the initial concentration of peptide solution in the donor chamber (mM). 

The ER was the ratio between the Papp value in the secretory (BL–AP) direction and the Papp value in the absorptive (AP–BL) direction (Equation (2)):

(2) $$\mathrm{ER}=\frac{Papp_{\left(BL-AP\right)}}{Papp_{\left(AP-BL\right)}}$$

Four inhibitors, including transport inhibitors cytochalasin D (a cell tight junction disruptor, 0.5 μg/mL), wortmannin (transcytosis inhibitor, 500 nM), sodium azide (ATP synthesis inhibitor, 10 mM), and Gly-Pro (transport peptide PepT 1 substrate, 10 mM) were applied to identify the transport routes of the peptide. After 120 min, the concentration of the peptide was determined in the basolateral samples [6].

### 3.6. LC-MS Analysis

Analysis of AP and BL samples gathered after the transport of WGAPSL across Caco-2 cells was performed by Agilent LC-MS (6420 Triple Quad, Waters, Japan). A symmetrical 4.6 × 250 mm C_18_ column (GL Sciences, Tokyo, Japan) was used for fragments separations. The analysis was done in the MS Scan mode, and the injection volume was set to 20 μL. The peptide was applied in formic acid as an organic regulator to improve the MS signal of the peptide. Solvents A and B were formic acid/milli Q water (0.1:100, v/v) and formic acid/acetonitrile (0.1:100, v/v). For WGAPSL, the sample elution was run at 0.7 mL/min using a linear gradient of solvent B in A from 18% to 43% for 25 min, 43–100% for 5 min, and 100–18% for 10 min to equalize the column. The accumulation and ionization conditions in the ion trap were optimized automatically, and the target mass of the molecular ion was as follows: *m*/*z* 630.633 (WGAPSL).

Analysis was performed using the positive detection mode. The gas temperature was 300 °C. The nebulizer pressure was set at 15 psi with a gas flow of 11 L/min and capillary voltage of 1400 V.

A standard curve was prepared with a synthetic peptide diluted in HBSS, and the peptide concentrations were 0–3.9 μM. The curve was analyzed as indicated above for the BL samples. Relying on the fit, the following curve was selected: y = 5 × 10^9^x − 461,920 (R^2^ = 0.9955).

### 3.7. Statistical Analysis

All data are expressed as means ± SE. Differences among groups were determined by one-way ANOVA analysis. The 95% confidence level was chosen to define statistical significance.

## 4. Conclusions 

The transport mechanism of the soybean-derived peptide WGAPSL, with cholesterol-lowering activity, was investigated. Furthermore, its degradation fragments at the AP and BL sides of Caco-2 cells during transport were analyzed by LC-MS. The results suggest that WGAPSL survived the action of intestinal peptidases and crossed the mucus layer to be absorbed intact through intestinal epithelium in vivo. The significant promoting effect of cytochalasin D on the transepithelial transport of WGAPSL suggests that paracellular transport was the main important mechanism of its transport through Caco-2 cells. This study evidences for the first time the absorption of the cholesterol-lowering peptide from soybean protein by the Caco-2 cell line. A further study will be carried out to investigate the cholesterol-lowering activities of peptide fragments released due to the hydrolysis of intestinal peptidase.
